# Eye closure enhances dark night perceptions

**DOI:** 10.1038/srep10515

**Published:** 2015-05-27

**Authors:** Stefan Brodoehl, Carsten M. Klingner, Otto W. Witte

**Affiliations:** 1 Hans Berger Department of Neurology, University of Jena, Germany; 2 Brain Imaging Center, University of Jena, Germany

## Abstract

We often close our eyes when we explore objects with our fingers to reduce the dominance of the visual system over our other senses. Here we show that eye closure, even in complete darkness, results in improved somatosensory perception due to a switch from visual predominance towards a somatosensory processing mode. Using a tactile discrimination task and functional neuroimaging (fMRI) data were acquired from healthy subjects with their eyes opened and closed in two environments: under ambient light and in complete darkness. Under both conditions the perception threshold decreased when subjects closed their eyes, and their fingers became more sensitive. In complete darkness, eye closure significantly increased occipital blood-oxygen-level-dependent (BOLD) activity in the somatosensory and secondary visual processing areas. This change in brain activity was associated with enhanced coupling between the sensory thalamus and somatosensory cortex; connectivity between the visual and somatosensory areas decreased. The present study demonstrates that eye closure improves somatosensory perception not merely due to the lack of visual signals; instead, the act of closing the eyes itself alters the processing mode in the brain: with eye closure the brain switches from thalamo-cortical networks with visual dominance to a non-visually dominated processing mode.

It is well known that engagement of the visual system may alter perception in other sensory systems. The first written reference to this intuitive concept was provided by Aristotle in “Metaphysics”, where he wrote that *we prefer seeing (one might say) to everything else*. In daily life, the simultaneous presentation of visual and auditory stimuli can suppress auditory information, a phenomenon known as the Colavita effect^1^. Similar observations have been made for other sensory modalities in humans^1^,^2^ and many other species. For example, seeing lip movements alters speech recognition (McGurk effect^3^) as well as the localization of the sound source (ventriloquist illusion^4^). Crossmodal interactions from the visual system, such as watching another person being touched, may also activate the somatosensory cortex^5^. Occasionally, vision also enhances the perception of non-visual stimuli, most likely via a shift of spatial attention and crossmodal interactions^6^. Conversely, visual deprivation, such as observed in prisoners of war over months and in healthy subjects over days, hours and minutes, improves specific aspects of tactile, auditory and gustatory perception^7^,^8^,^9^.

The present study characterizes how vision alters perception in non-visual sensory modalities by studying interactions between the visual and somatosensory system. We hypothesized that deprivation of visual signals, which was achieved by eye closure, would result in an improved processing within the somatosensory system, namely via an increased cooperation and processing of the sensory thalamus and somatosensory cortices. To test this hypothesis the sensory perception thresholds was determined in 16 healthy subjects who had to alternately open and close their eyes.

## Methods

### Subjects

16 right-handed subjects (10 females, mean age 23.1 ± 1.54 years) without any history of neurological or psychiatric disease participated in the study (all subjects were examined by a neurologist on the first day of investigation). Informed written consent was obtained in accordance with the Declaration of Helsinki and the local ethics committee (Friedrich-Schiller-University Jena, Germany) from all subjects. The experimental protocol was approved by the local ethics committee as well. All participants were right-handed as determined using a standardized inventory, with a lateralization index greater than 70% as an inclusion criterion. All subjects were asked to refrain from caffeine at for least 6 hours and from alcohol for at least 24 hours before the examination.

Informed written consent was obtained in accordance with the Declaration of Helsinki and the local ethics committee (Friedrich-Schiller-University Jena, Germany) from all subjects. The experimental protocol (including the functional magnetic resonance imaging experiments described below) was approved by the local ethics committee.

### Current perception threshold (CPT)

Differences in the perception thresholds were determined between conditions with eyes open and eyes closed in a standardized illuminated room, and in complete darkness.

Starting in a relaxed sitting position with closed eyes (illuminated), a 40-Hz monophasic wave pulses starting at 0.5 mA was applied to the right index finger using a clinical neurostimulator (Digitimer Constant Current Stimulator model DS7A, Digitimer Ltd, Welwyn Garden City, Hertfordshire, AL7 3BE, England). The current intensity was slowly increased until each subject detected the stimulus. The procedure was repeated up to 20 times until we achieved a constant baseline CPT.

Starting with opened eyes, the subjects alternately opened and closed their eyes for 5 minutes repeatedly (5 times each, total time 50 minutes). Within each block, the CPT was determined every 30 seconds.

Next, the measurements were repeated in complete darkness. Volunteers had to wear completely darkened goggles, the room was darkened, and each volunteer confirmed that during the entire examination, no gleam of light was noticed. After 10 minutes of dark adaption and the determination of a baseline, 5 CPT blocks of eyes opened and eyes closed (5 minutes each) were performed, and CPTs were determined every 30 seconds. The investigator verbally gave instructions to open/close the eyes.

### Mechanical detection threshold (MDT)

In addition to the CPT, mechanical detection threshold (n = 10) in complete darkness was determined by a standardized set of modified von Frey hairs (Optihair_2_-Set, Marstock Nervtest, Germany). These hairs apply pressure between 0.25 and 512 mN (grading by a factor of 2); for methodical details please refer to Rolke *et al.*^10^. The von Frey hairs were applied on the skin of the right backhand for 1-2 s in a region of uniform size and shape (spot with a diameter of 0.5 mm) without any visible body hair. 5 threshold determinations were alternately acquired starting with descending from a supra-threshold intensity and then ascending from an infra-threshold intensity (method of limits). The final threshold was the geometric mean of these 5 series. A block of 5 minutes eyes closure followed by 5 minutes of opened eyes was repeated 4 times (total time 40 minutes); within each block the MDT was determined 2 times; mean and standard error of all blocks (closed vs. opened eyes) were calculated.

### MRI Recordings

All experiments were performed using a 3.0-T MR scanner (Trio, Siemens, Erlangen, Germany) to obtain echo-planar T2*-weighted image volumes (EPI) and transaxial T1-weighted structural images. The high-resolution T1-weighted structural images had a voxel size of 1 × 1 × 1 mm^3^ to enable precise anatomical localization. fMRI experiments were performed in complete darkness, and the volunteers had to wear completely darkened goggles. The investigator verbally gave instructions to the subjects via earphones to alternately open and close their eyes.

Three different fMRI experiments were performed. According to a standardized protocol, no subject reported fatigue or unpleasant sensations during the experiment.

In the first fMRI experiment, a block design was used. Starting from the eyes closed condition, the subjects had to alternately open and close their eyes every 27 seconds (20 blocks each, total time of less than 20 minutes). In total, 600 EPI images (voxel size = 3 mm × 3 mm × 3 mm, repetition time = 2.52 s, TE = 35 ms; 40 transaxial slices, including the entire cerebrum and cerebellum) were acquired.

In the second experiment, we used an identical block design as in the first experiment, but within each block, tactile stimulation was applied to fingers 1 – 5 of the right hand. The stimulation was delivered using balloon diaphragms driven by compressed air. Each stimulus lasted for 100 ms (20 ms rise time, 30 ms plateau and 50 ms return to baseline pressure) and was presented in an event-related regime. To avoid systematic errors in the hemodynamic response function estimation, the stimulus time was randomized between 8.7 and 15.8 seconds after the block began. The timing of the stimulus presentations was externally controlled by the MRI scanner and was synchronized to image acquisition.

In the third experiment, resting-state fMRI was recorded starting with the eyes closed condition for 9 minutes, followed by the eyes opened condition for another 9 minutes. For each block, 240 EPI images (voxel size = 3 mm × 3 mm × 3 mm, repetition time = 2.52 s, TE = 35 ms; 40 transaxial slices, including the entire cerebrum and cerebellum) were recorded.

### Data analysis

Data analysis was performed on a PC using MATLAB (Mathworks, Natick, MA) and SPM8 software (Wellcome Department of Cognitive Neurology, London, UK, http://www.fil.ion.ucl.ac.uk/spm). For each subject, all images were realigned to the first volume using six-parameter rigid-body transformations to correct for motion artifacts^11^,^12^. The images were co-registered with the corresponding anatomical (T1-weighted) images of the subject, re-sliced to correct for acquisition delays (referenced to the tenth slice only in the event-related design), normalized to the Montreal Neurological Institute (MNI) standard brain^13^ to report MNI coordinates and smoothed using a 6-mm full-width-at-half-maximum Gaussian kernel.

### fMRI analysis

Multiple regression analysis using a general linear model was performed to obtain statistical parametric maps calculated for the somatosensory stimulation. The fMRI signal time courses were high-pass filtered (128 s) and modeled as an experimental-stimulus onset function convolved by the canonical hemodynamic response function (low-pass filter). Two contrasts of interest were examined, resulting in two t-statistical (paired t-test) maps (eyes closed > opened and closed < opened for the first fMRI experiment, stimulation while eyes closed > opened and stimulation while closed < opened for the second fMRI experiment). Individual results were projected onto their respective co-registered high-resolution T1-weighted 3-D data set. The anatomical localization of the activated areas was analyzed with reference to the standard stereotaxic atlas and was mapped using the anatomical toolbox of the SPM program^14^,^15^ ( http://www.fz-juelich.de/ime/spm_anatomy_toolbox). The resulting statistical maps were thresholded by the FDR.

To evaluate the shape, timing (time to peak) and magnitude (height and full-width at half-maximum) of the task/stimulus-evoked hemodynamic response in the second fMRI experiment, we extracted the time course of the cluster of the highest t-values within the primary somatosensory cortex (−44 × −28 × 62 including 26 surrounding voxel). We performed a least-squares fitting of the experimental signal time courses with an inverse logit function as previously described by Lindquist and Wagner^16^:


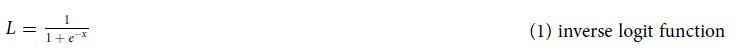



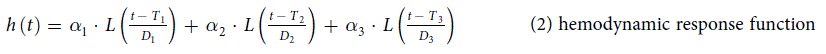



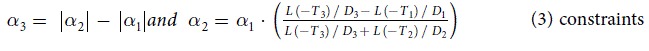


Start parameters (D1 = −1.834, D2 = −0.6314, D3 = −3.016, T1 = 4.358, T2 = 2.715, T3 = 4.516, α1 = 5.143) were determined by fitting the model to the SPM built-in hemodynamic response function. All individual BOLD time courses were fitted to the model. To evaluate the quality of the curve fit, the R-Square and root mean squared error (RMSE) are listed.

### Functional connectivity analysis of the resting-state fMRI

Changes in functional connectivity within the somatosensory, visual and thalamic network induced by eye closure were investigated in the resting state. We were particularly interested in the analysis of the bilateral SI and SII, V1-5 and thalamus. The anatomical location of each area was determined by referencing the standard stereotaxic atlas and was mapped using the SPM program anatomical toolbox ( http://www.fz-juelich.de/ime/spm_anatomy_toolbox)^4^. The point of maximum activation strength within the left and right SI and SII (along with its 26 neighboring voxel) was selected from the group analysis of the stimulation task of the second fMRI experiment. For the visual areas V1-V5, we employed the areas of the highest group t-values of the first fMRI experiment. For the thalamus, we defined clusters of 50 voxels for the right- and left-sided prefrontal zones, the motor zone, the somatosensory zone and the parieto-occipital zone as provided by Behrens and colleagues^17^.

The resting-state data from these identified regions of interest (ROIs) were extracted, and cluster-specific time series were then estimated by averaging the time series of all voxels within a cluster. Several sources of variance were removed from the data using linear regression: (1) six parameters obtained by rigid body correction of head motion, (2) a signal from a ventricular region of interest and (3) a signal from a region centered in the white matter^18^. All signal intensity time courses were band-pass filtered (0.01 < f < 0.1 Hz) to reduce the effects of low-frequency drift and high-frequency noise. The Pearson correlation coefficients were computed between the clusters as previously described and then converted to z-values by the Fisher r- to z-transformation. To test for differences between the two resting-state scans, we used paired t-tests to compare the z-map of the first scan (eyes closed) with the corresponding z-map of the second scan (eyes opened) for each subject. Findings in the resulting group map were considered significant at p < 0.05 (FDR-corrected cluster threshold).

### Conditional Granger causality analysis of resting-state fMRI

Conditional Granger causality analysis (CGCA) is an approach used to explore the dynamic causal relationship between time series^19^. This approach has been widely used in previous fMRI studies^20^,^21^,^22^,^23^. In this study, the CGCA was performed using the toolbox implemented by Seth^24^. The detailed theory behind Granger causality has been previously described^19^,^24^,^25^. However, a brief introduction of the Granger procedure is provided below.

The fundamental idea is that a time course X1 causes the time course X2 if the knowledge of X1 helps to predict the future of X2. In a linear regression model of X1 and X2, X1 causes X2 if the inclusion of past observations of X1 reduces the prediction error of X2, compared to a model that includes only previous observations of X2. The bivariate autoregressive model can be written as


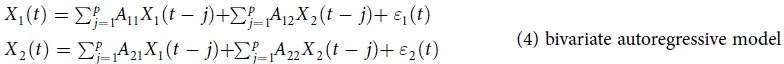


where p is the maximum number of lagged observations included in the model. The matrix, A, contains coefficients of the model and represents the residuals. The magnitude of the interaction between X2 and X1 can be measured using the following equation:


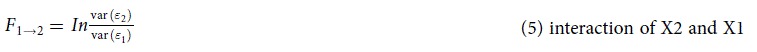


This model can be generalized in the context of multiple additional variables X3….Xn and is known as “Conditional Granger causality”^24^.

Using the Akaike information criterion, we found that two was the optimum number of time-lags (p) to be included in the analysis. We used 6 time courses of 6 different ROIs in the analysis, including the left-sided SI and SII, the somatosensory and occipital zone of the thalamus and V1 and V4 as defined by the estimation of the functional connectivity. The influence that one of the selected brain regions exerts over another region was estimated for both resting-state scans and was then analyzed for significant differences using a paired t-test. In addition, the autonomy of each selected brain area was estimated and compared between the scans. The autonomy of a variable describes the extent that its own past influences its future and that these predictions are not accounted for by external variables^24^. The autonomy is also considered to be the degree of self-determination of self-causation^24^,^26^,^27^. Findings were considered significant at p < 0.05 (FDR-corrected).

## Results

**Figure 1:**
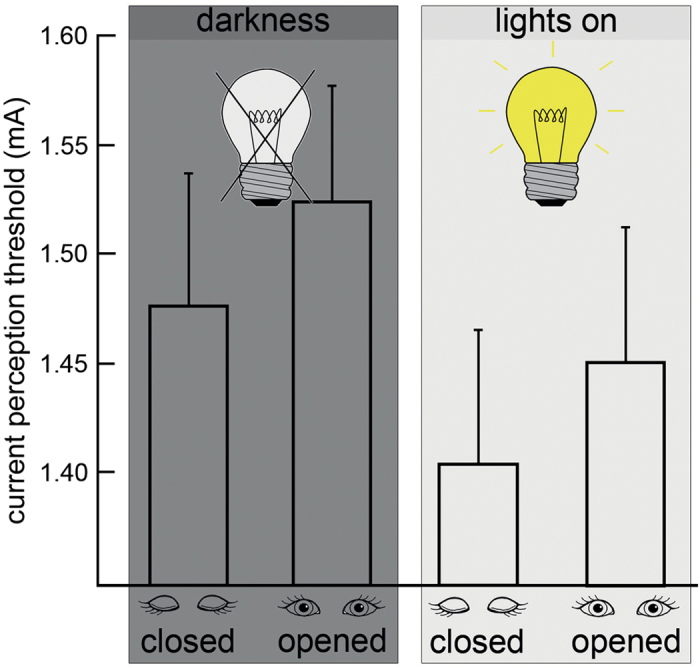
Impact of eye closure on current perception threshold (CPT). Electrical stimulation was applied to the right index fingers of 16 healthy subjects (10 females, mean age 23.1 ± 1.54 years). The thresholds were significantly decreased (higher sensitivity) when the eyes were closed in standard (normal environment light [right], paired t-test (2-tailed) p ≤ 0.004) and control (completely darkened room [left], paired t-test (2-tailed) p ≤ 0.001) conditions. A two-way ANOVA (to correct for inter-individual variance of the CPT, normalized CPT was calculated by dividing individual CPTs by the CPT in lights on / eyes opened) using two independent factors (lights on / darkness and eyes opened / closed) revealed a significant main-effect of eyes opened / closed and CPT (p ≤ 0.05). There was no significant main-effect for environment lights on / darkness and no significant interaction of eye state and environment. Error bars indicate the mean standard deviation. Please refer to Table 1 for individual results and group statistics.

Determination of the sensory perception thresholds showed that the subjects were significantly more sensitive to somatosensory stimuli with closed than with open eyes. As a control experiment, this sequence was repeated in complete darkness. Unexpectedly, eye closure in complete darkness also resulted in an enhancement of tactile sensory sensitivity similar to that observed in light (Fig. 1, Table 1). This finding could be replicated by determining the mechanical detection threshold (MDT) instead of the CPT (closed eyes: MDT mean 0.57 ± 0.32 N, opened eyes: MDT mean 0.83 ± 0.47 N, difference was significant at p ≤ 0.017 (two-tailed paired t-test))(Figure S3 in Supplementary Materials).

These observations suggested that improved somatosensory perception after eye closure is caused by a switch in the traffic mode of the brain independent of visual information: the different processing modes are activated by eye closure/eye opening even when performed in complete darkness. This implies that the brain centers, which govern eye opening and eye closure, have direct effects on information processing in the visual and somatosensory brain areas. To unmask the underlying structures and functional mechanisms, further electroencephalography (EEG, please compare the description of the methods in the supplementary) and functional magnetic resonance imaging (fMRI) data were acquired.

Investigations were performed on the same subjects as the psychophysical experiments. For all of the experimental sessions, the EEG and MRI scanner room was completely darkened, and the subjects were blindfolded. Consistent with recent reports^28^,^29^, closure of the eyes induced occipital alpha EEG activity also in complete darkness (data not shown).

**Figure 2:**
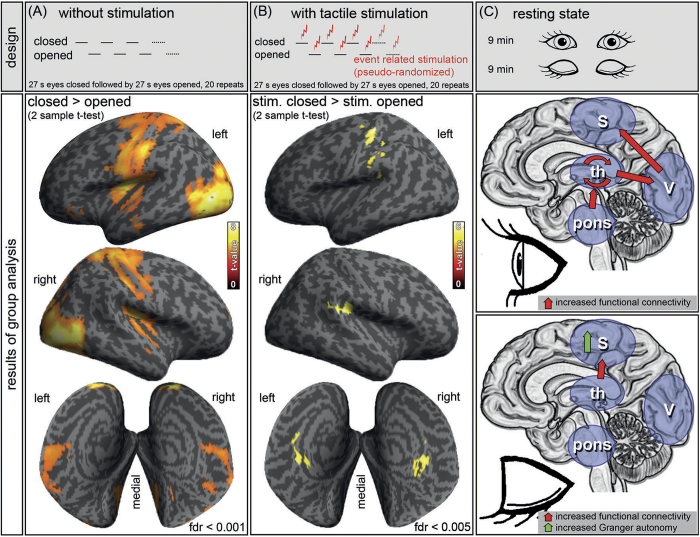
Impact of eye closure on resting brain activity (**A**), event-related BOLD activation (**B**) and resting state activity (**C**) in complete darkness. Resting brain activity was determined as difference between activity with eyes closed as compared to eyes open (**A**). Tactile stimulation was performed at the right hand with closed and open eyes (**B**). Changes in functional and effective connectivity were determined with eyes open, and eyes closed (**C**). At the given thresholds (and corrections), no significant activations for closed < opened and stimulation closed < stimulation opened were observed. All fMRI experiments were performed in complete darkness. To ensure constant alertness, an EEG was continuously recorded while the participants (N = 16, 10 females, mean age 23.1 ± 1.54 years) were lying in the MRI scanner.

To determine the impact of eye closure on brain activity, the subjects lay in the MRI scanner and alternately opened and closed their eyes every 27 seconds (Fig. 2, left). Eye closure was associated with increased BOLD activity in large cortical areas, including the secondary visual areas and the primary and secondary somatosensory cortices (Table S1 in Supplementary Materials). The primary visual cortex was not affected (Figure S1 in Supplementary Materials).

Next, we investigated how eye closure in darkness altered task-induced brain activity associated with somatosensory processing (Fig. 2, middle). Again, the subjects lay in the scanner and alternately opened and closed their eyes. In addition, a tactile stimulation to fingers 1 – 5 of the right hand was provided using a pneumatic device. Stimulation occurred in a pseudo-randomized order, with each train of stimulations lasting for two seconds. As expected from the psychophysiological data, which showed improved somatosensory perception with closed eyes, the functional activation (as determined by a random effect analysis) within the contralateral primary somatosensory cortex and both secondary somatosensory cortices increased in the eyes closed condition (Table S2 in Supplementary Materials). To characterize changes in the timing and shape of the local blood-oxygen-level-dependent (BOLD) response, the hemodynamic response function (HRF) was fitted. This revealed increased amplitudes and a faster peak following tactile stimulation with eyes closed within the primary somatosensory cortex (Figure S2 in Supplementary Materials).

**Figure 3:**
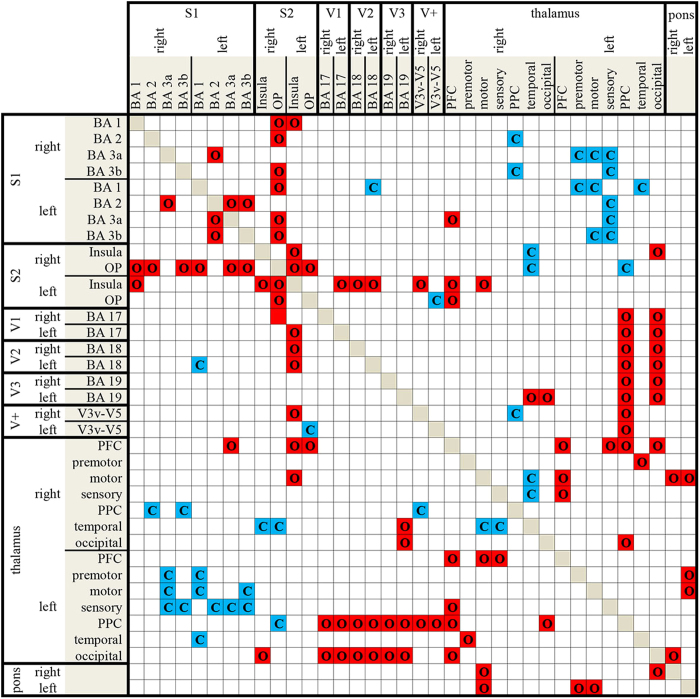
Results of the functional connectivity analysis. BA - Brodmann area, V1-5 visual cortices, C (blue) - increased connectivity when the eyes are closed, O (red) - increased connectivity when the eyes are opened. Group analysis was considered significant when p ≤ 0.05.

To study the functional interaction of visual and somatosensory brain areas with eye closure and opening in darkness, the functional and effective connectivity of spontaneous brain activity in the resting-state fMRI were examined (Fig. 2, right and Fig. 3). Resting-state fMRI was performed on the same subjects as before. The subjects lay still in complete darkness for 9 minutes with opened and then with closed eyes. In the eyes open condition, functional connections between the thalamus and primary visual cortex, between the primary visual and the primary and secondary somatosensory cortices and within different subregions of the thalamus were observed. Eye closure increased the functional connectivity between the thalamus and somatosensory cortex and decreased coupling between the thalamus and visual cortex. Furthermore, the eyes closed condition was accompanied by a reduced coupling between the brain stem and visual thalamus. Effective connectivity (as measured using the Granger autonomy index) revealed an enhanced autonomy of the somatosensory system with closure of the eyes (significant at p < 0.05, false discovery rate [FDR]-corrected).

## Discussion

In summary, eye closure in complete darkness increases occipital alpha-synchronization in the EEG, and BOLD activity in the somatosensory cortex and higher order visual areas. This is accompanied by increased connectivity of somatosensory thalamus and somatosensory cortex, and by decreased connectivity between visual and somatosensory cortices. BOLD activation induced by somatosensory stimulation is increased with closed eyes (both in spatial expansion and amplitude) in task-relevant somatosensory cortical areas. These findings are accompanied by superior perceptional performance.

Importantly, we ensured that the subjects experienced complete darkness in the absence of exposure to any residual light, which sometimes occurs in many other paradigms involving blindfolding^8^,^28^,^29^,^30^. Strict conservative corrections for the fMRI (all group results were FDR-corrected) and psychophysiological data (the paired t-test was regarded significant at p ≤ 0.001), were used to adjust for potential statistical errors.

Rhythmic 10-s EEG activity in posterior brain regions during the resting state with closed eyes (alpha activity) is well known. Reduction of this activity with opening of the eyes has been documented since the invention of the EEG by Hans Berger and is called the *Berger effect*^31^. The alpha rhythm is often regarded as a resting or “idling” activity of the cortex, whereas more recent studies suggest that it also aids neurons in distributed networks to effectively activate and integrate different brain structures^32^,^33^. The occipital alpha activity is associated with a metabolic deactivation of the underlying cortex^34^. Until recently, it was unclear whether the Berger effect depends on vision or is also present in darkness. As shown in our study, closing the eyes in complete darkness increases BOLD activity in the somatosensory cortex and higher visual areas but not within the primary visual cortex (Figure S1 in Supplementary Materials), and increases occipital alpha-synchronization^28^,^29^. Taken together, the observed changes in electrophysiological (EEG) and BOLD brain activity are expected to increase somatosensory perception with eye closure as observed in the present study.

A previous study reported an increased skin conductance in eyes-opened condition^35^. To exclude an impact of such factors on our results, we performed a tactile task in complete darkness using a standardized set of von Frey hairs^10^. This confirmed a significantly improved tactile detection threshold with eyes closure (Figure S3 in Supplementary Materials). In our study we did not systematically include sham stimuli as suggested in recent methodology of psychophysical testing^36^,^37^. This may result in some false positive threshold perceptions; however, it cannot explain the reproducible differences between the tested conditions.

It is well known that perception in different modalities, e.g., hearing or touch, may be altered by vision. Merabet *et al.* reported changes in cortical processing in response to tactile stimulation in blindfolded subjects over the course of days and recovery to baseline activity within 24 hours^8^. Similar findings were described down to a blindfold period of 45 minutes^38^,^39^. Some recent studies questioned these results^40^,^41^. Visual deprivation lasting from 10 to 110 minutes had no effect on the performance in a grating orientation task, whereas the discrimination appeared slightly superior in an opened eye condition. With the exception of the study by Wong *et al.*^41^, these studies did not consider different baseline conditions such as opened or closed eyes before blindfolding^40^. The described studies differ in two important aspects from the present study. Firstly, we did not investigate visual deprivation but the impact of eye closure (in complete darkness). Secondly, grating orientation discrimination involves a distributed network of multisensory and integrative brain areas^42^ which may be different from the current and mechanical perception threshold described in this study; it is conceivable that eye closure may even impede processing of sensory information that employs multisensory integration^41^.

In our study, changes in the perception threshold and brain activity occurred within a few seconds (fMRI experiments with and without stimulation during the eyes closed period, 27 seconds for each block) or a few minutes (5 minutes of eyes closure and eye opening to measure the current perception threshold and 9 minutes for the fMRI resting-state). We rapidly open and close our eyes several thousand times a day, and unlike in cross-modal plasticity, which has been previously described in other studies^7^, no evidence for any persistent effects was found in the present study. Relaxed eye closure is a passive state that is achieved by the relaxation of the levator palpebrae superioris muscle with little or no activity of the orbicularis oculi; however, continuous activation of the levator muscle is required to hold the eyes open^43^. At the brainstem level, eye closure enhances reflex responses (e.g., the acoustic reflex threshold), and this effect is most likely mediated via the reticular system^44^. Eye movements may have an impact on neuronal activity in the thalamus and the visual cortex^45^,^46^,^47^,^48^. However, there are no indications that eye-drifts vary significantly between opened and closed eyes in complete darkness^45^. Therefore, our data are best explained by a functional link between the brainstem and thalamus that gates competing sensory systems depending on the activity of the oculomotor system (Fig. 2, right).

The present study supports the following model of brain activity^49^: with open eyes, a gain control (implemented by increased connectivity between the thalamus, pons and visual cortex) sets the visual channel to a dominant mode (Fig. 2, right), which enslaves other sensory cortices. This visual processing mode is diminished by eye closure, with light as well as in the dark. Eye closure will thus rapidly unmask salient connections within the thalamus and somatosensory cortex, while crossmodal cortico-cortical pathways connecting the visual areas with non-visual areas are masked^50^. This model is supported by the existence of anatomical long-range pathways that connect the primary sensory cortices with other sensory or multimodal cortices^51^ and adaptable subcortical pathways^52^.

Open eyes represent the native condition in awake humans. Thus, a high level of connectivity between the visual and non-visual areas and multisensory integration should be an advantage in daily life^53^. The switch to a non-visually dominated processing mode may either be preformed in the brain, or learned as a condition of focused attention. Whether the phenomenon described in our study is exclusive for the somatosensory system or (what appears to be more intuitive) is relevant for all non-visual sensory systems requires further studies.

## Additional Information

**How to cite this article**: Brodoehl, S. *et al.* Eye closure enhances dark night perceptions. *Sci. Rep.* **5**, 10515; doi: 10.1038/srep10515 (2015).

## Supplementary Material

Supplementary Information

## Figures and Tables

**Table 1:** Results of the electrical stimulation of the right index fingers of the subjects under conditions of (A) light / eyes closed, (B) light / eyes opened, (C) darkness / eyes closed and (D) darkness / eyes opened.

			**lights on**				**darkness**			
			***(A)***		***(B)***		***(C)***		***(D)***	
			***closed***		***opened***		***closed***		***opened***	
#	**age (years)**	**sex**	**mean (mA)**	**s**	**mean (mA)**	**s**	**mean (mA)**	**s**	**mean (mA)**	**s**
1	21	m	2.00	0.11	2.03	0.09	2.01	0.21	2.18	0.05
2	21	m	1.75	0.18	1.98	0.18	1.65	0.13	1.69	0.09
3	22	w	2.01	0.11	2.06	0.09	1.95	0.21	2.13	0.05
4	22	m	1.73	0.18	1.85	0.18	1.66	0.13	1.70	0.09
5	22	w	1.18	0.04	1.15	0.05	1.17	0.06	1.21	0.07
6	23	w	1.84	0.05	1.90	0.02	2.27	0.03	2.32	0.04
7	23	w	1.10	0.01	1.13	0.03	1.13	0.02	1.15	0.03
8	23	w	1.03	0.03	1.08	0.04	1.06	0.05	1.10	0.03
9	23	w	1.17	0.04	1.19	0.05	1.15	0.06	1.18	0.07
10	23	m	1.13	0.03	1.18	0.03	1.14	0.02	1.13	0.04
11	23	w	1.23	0.02	1.20	0.05	1.41	0.03	1.44	0.04
12	23	m	1.12	0.03	1.14	0.03	1.14	0.02	1.10	0.04
13	24	w	1.24	0.02	1.25	0.05	1.40	0.03	1.46	0.04
14	24	w	1.83	0.05	1.92	0.02	2.22	0.03	2.27	0.04
15	26	w	1.04	0.03	1.10	0.04	1.11	0.05	1.14	0.03
16	27	m	1.08	0.01	1.06	0.03	1.16	0.02	1.19	0.03
MW	23.1±1.54		1.41±0.06		1.45±0.06		1.48±0.07		1.52±0.05	
	paired t-test		p ≤ 0.004				p ≤ 0.001			

Each value represents the mean value of 5 blocks (5 measurement per block). (s) Standard deviation. A paired two-tailed t-test was performed on the current perception thresholds in mA (last row).
