# Supplementary material for: Eye closure enhances dark night perceptions
Source: Sci Rep. 2015 May 27;5:10515. doi: 10.1038/srep10515 (PMC4444970; doi:10.1038/srep10515)
Supplement: Supplementary Information [file srep10515-s1.pdf]

Supplementary Information

Title: **Eye closure enhances dark night perceptions**

Authors: Stefan Brodoehl[1,2]*; Carsten Klingner[1,2] and Otto W. Witte[1,2]

Affiliations:

[1] Hans Berger Department of Neurology, University of Jena, Germany

[2] Brain Imaging Center, University of Jena, Germany

*Corresponding author: stefan.brodoehl@med.uni-jena.de

This file includes:

Figures S1-S3

Tables S1-S2

Supplementary Materials:

EEG Recordings

The EEG was recorded from 21 electrodes that were referenced to Cz using a standard EEG cap (Easy Cap, Falk Minow Services Germany) based on the international 10-20 system. All electrode impedances were maintained at < 5 kΩ; after filtering (0.1 – 100 Hz), the EEG was sampled at 500 Hz. In this study, only data obtained from 12 electrodes were processed: F3, Fz, F4, C3, Cz, C4, P3, Pz, P4, O1, Oz and O2. These electrodes were selected because they were mainly situated in the regions of the somatosensory and visual cortices.

Additional information to the current percept threshold

Differences in the perception thresholds were determined between conditions with eyes open and eyes closed in a standardized illuminated room, and in complete darkness.

Starting in a relaxed sitting position with closed eyes (illuminated), a 40-Hz monophasic wave pulses was applied to the right index finger using a clinical neurostimulator (Digitimer Constant Current Stimulator model DS7A, Digitimer Ltd, Welwyn Garden City, Hertfordshire, AL7 3BE,

England). Electrodes were placed at the base medial and middle phalanx of the index finger (palmar). The contact area was prepared by cleaning with a diluted ethanol solution; electrodes were fixed using a conductive and adhesive paste (Ten20®, Weaver and Company, 565 Nucla Way Unit B, Aurora, CO 80011). The ground electrode was placed on the lateral backside of the right hand.

The current intensity was slowly increased starting at 0.5 mA by manually turning a control wheel until each subject detected the stimulus. To test for false positive perceptions we varied the time gap between consecutive stimuli within 30 seconds. Investigator and stimulator were placed behind the participants, therefore they could neither visually nor acoustically anticipate the stimulus procedure.

Wave impulses (length and frequency) were triggered automatically using a stimulation software (Presentation®, Neurobehavioral Systems, Inc., 15 Shattuck Square, Suite 215, Berkeley, CA 94704-1151). The procedure was repeated up to 20 times until we achieved a constant baseline CPT. A baseline CPT was defined when 5 consecutive measurements did not differ in range more than 10%. In average 12 measurements were performed until a stable baseline was achieved.

Starting with opened eyes, the subjects alternately opened and closed their eyes for 5 minutes repeatedly (5 times each, total time 50 minutes). Within each block, the CPT was determined every 30 seconds.

Next, the measurements were repeated in complete darkness. Volunteers had to wear completely darkened goggles, the room was darkened, and each volunteer confirmed that during the entire examination, no gleam of light was noticed. After 10 minutes of dark adaption and the determination of a baseline, 5 CPT blocks of eyes opened and eyes closed (5 minutes each) were performed, and CPTs were determined every 30 seconds. The investigator verbally gave instructions to open/close the eyes.

Fig. S1. Additional views of MRI BOLD response to electrical stimulation of the fingers. Electrical stimulation was applied to the right index fingers of 16 healthy subjects (10 females, mean age 23.1 ± 1.54 years).

Eye closure resulted in increased activity in the secondary and higher visual areas, whereas the primary visual cortex was not affected.

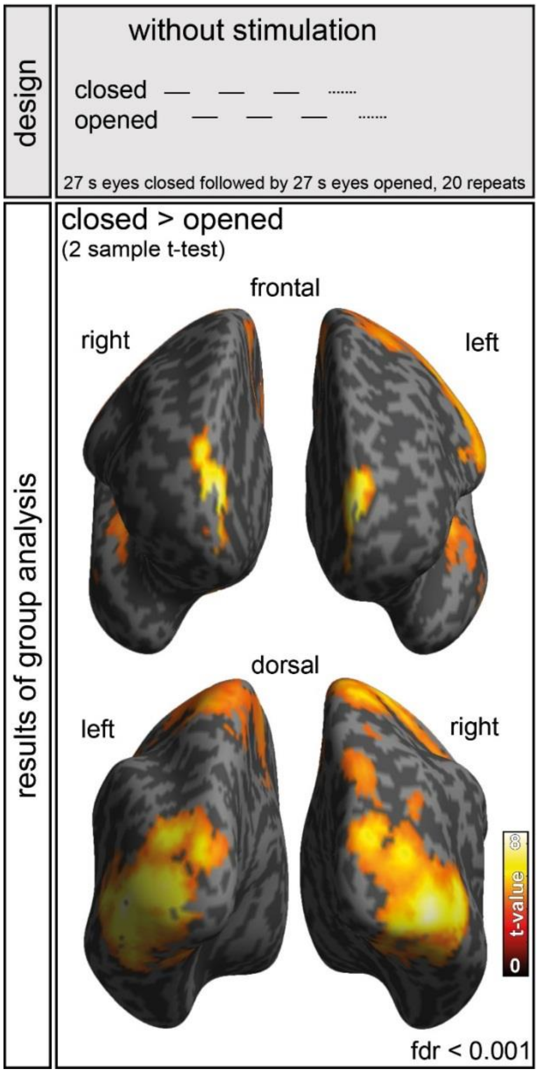

Fig. S2. Fitted time courses of the BOLD signal of a cluster with the highest t-values within the primary somatosensory cortex (MNI coordinates -44 × -28 × 62 and 26 surrounding voxels) in the second fMRI experiment (tactile stimulation was performed at the right hand with closed and open eyes). BOLD time courses of all subjects in response to stimulation with closed (blue) and opened (red) eyes were extracted (raw data) and fitted to an inverse logit function (fitted signal). For details and start parameters, please refer to the Methods in the Supplementary Materials. In the eyes closed condition, the fitted hemodynamic response function peaks earlier (ttp – time to peak) and is greater in shape (amplitude and full-width at half-maximum [fwhm]). To evaluate the goodness of the curve fit, the R-Square and root mean squared error (RMSE) are listed.

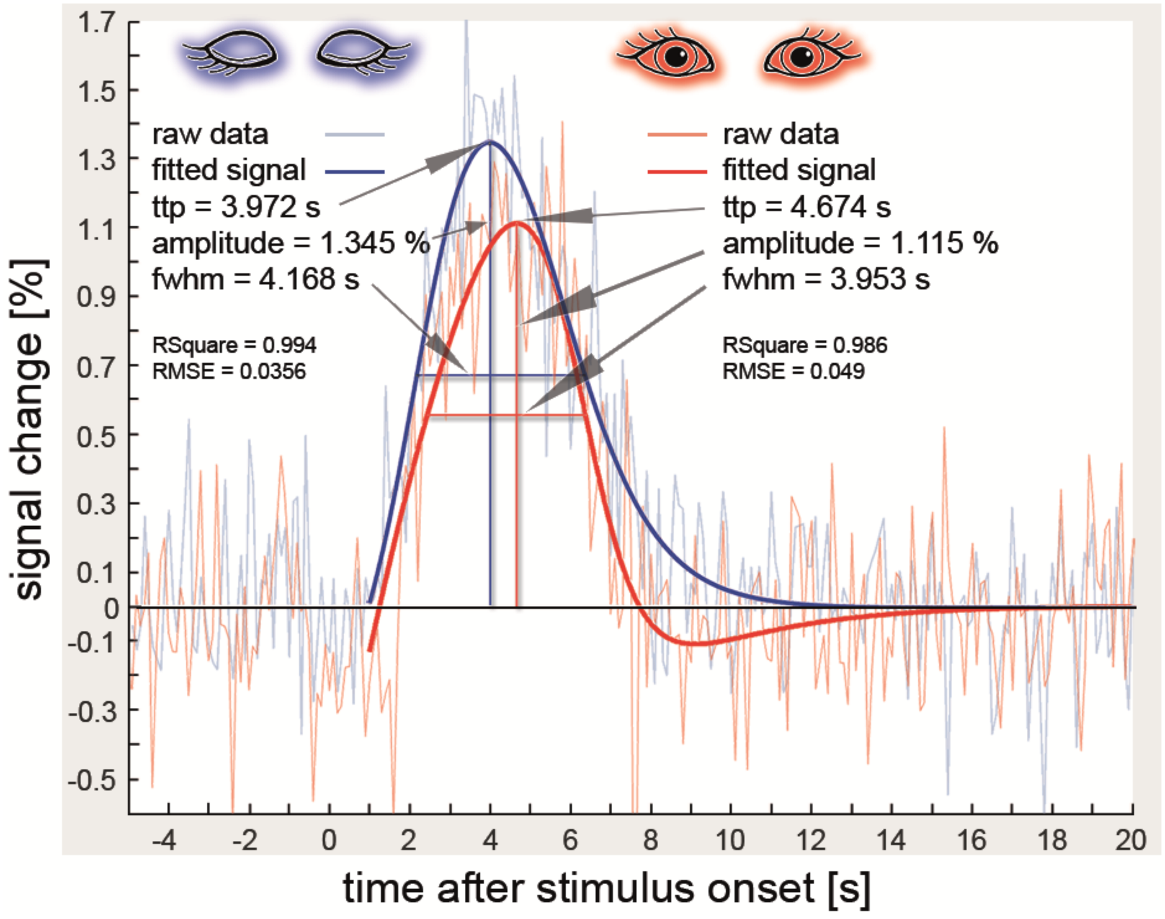

Fig. S3. Impact of eye closure in complete darkness on the mechanical detection threshold (MDT). Stimulation was applied with a standardized set of modified von Frey hairs. The thresholds were significantly decreased (higher sensitivity) when the eyes were closed (MDT mean 0.57 ± 0.32 N) compared to opened eyes (MDT mean 0.83 ± 0.47 N); the difference was significant at $p \leq 0.017$ (two-tailed paired t-test).

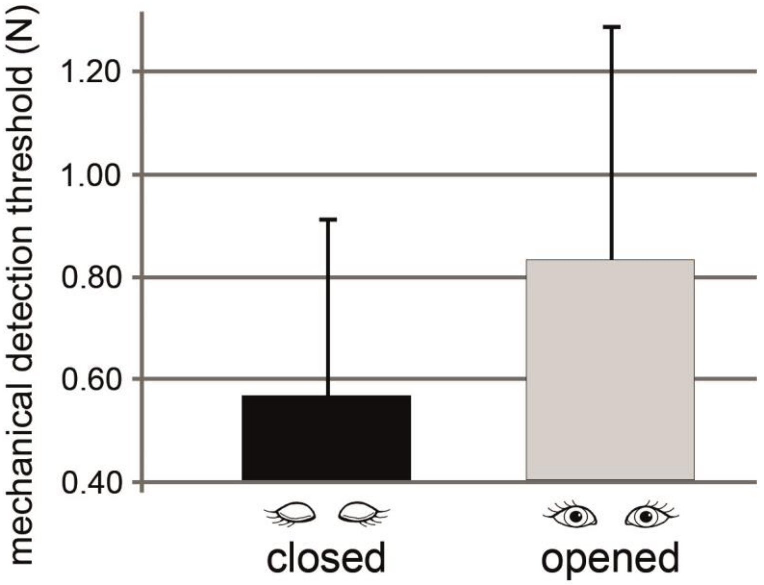

Table S1. Results of the random effect group analysis of the cortical activations for closed eyes > opened eyes. Clusters are sorted by the amount of significant voxels. Brodmann areas (BAs) are noted in decreasing order by voxel count. BAs 3, 2 and 1 are regarded as the SI, OP and insular overlap with the SII, respectively. BA 4 refers to the primary motor cortex, and BA 6 refers to the supplementary motor cortex. Visual areas 1– 5 are denoted by V1-5. The MNI coordinates of the highest (peak) t-values are noted. IPC - inferior parietal cortex, OP - operculum. % indicates the share of activation within whole cluster.

**Group analysis of cortical activation with closed eyes > opened eyes (corrected FDR < 0.001)**						
**Cluster (Voxels)**	Brodmann areas	hemisphere	peak at (MNI)	t-value		
			X Y Z	peak	mean	std
**1 (7049)**	6 (12%), OP (8.2%), 3b (5.6%), 4a (5.3%), 7 (4.5%), 5 (4.0%), 3a (3.2%), 2 (2.9%), IPC (2.6%), V4 (2.0%), 1 (1.7%), insular (1.5%), V3 (1.2%), V2 (1.2%), V1 (0.6%), V5 (0.5%)	left + right	51 -81 3	8.662	5.053	0.622
**2 (1591)**	V4 (8.4%), V3 (5.4%), V2 (5.2%), V5 (1.4%), IPC (1.4%), V1 (0.5%)	left	-51 -78 0	8.545	5.626	0.882
**3 (432)**	10, 11	left + right	3 63 6	7.901	5.791	0.931
**4 (39)**	45	left	-39 27 -12	5.069	4.663	0.210

Table S2. Results of the random effect group analysis of tactile stimulation of fingers 1 – 5 of the right hand for closed eyes > opened eyes. Clusters are sorted by the amount of significant voxels. BAs are noted in decreasing order by voxel count. BAs 3, 2 and 1 are regarded as the SI, OP and insular overlap with the SII, respectively. BAs 24 and 23 correspond to the ventral anterior and posterior cingulate cortices, respectively, and BA 32 corresponds to the dorsal anterior cingulate cortex. BA 4 refers to the primary motor cortex, and BA 6 refers to the supplementary motor cortex. The MNI coordinates of the highest (peak) t-values are noted. IPC - inferior parietal cortex, OP - operculum. % indicates the share of activation within the entire cluster.

**Group analysis of cortical activation in response to tactile stimulation of fingers 1 – 5 of the right-hand stimulation with closed eyes > stimulation with opened eyes (corrected FDR < 0.005)**						
**Cluster (Voxels)**	Brodmann areas	hemisphere	peak at (MNI)	t-value		
			X Y Z	peak	mean	std
**1 (640)**	1 (28.8%), 2 (14.8%), OP (12.0%), IPC 9.5%, 3b (5.2%), 4 (5.3%), 6 (4.0%)	left	-44 -28 62	5.916	4.700	0.376
**2 (495)**	24, 31, 32, corpus callosum	left + right	0 -26 30	5.362	4.534	0.291
**3 (306)**	OP (56%), IPC (28%)	right	64 -38 22	5.821	4.611	0.354
